# Effects of long-term biochar application combined with chemical fertilization on the nitrifier community in tobacco plantation soil

**DOI:** 10.3389/fmicb.2025.1720769

**Published:** 2025-12-11

**Authors:** Di Xu, Hang Luo, Tianyi He, Yang E, Junyi Gao

**Affiliations:** 1 National Biochar Institute, Shenyang Agricultural University, Shenyang, China; 2 Guizhou Tobacco Company in Bijie Company, Bijie, China

**Keywords:** biochar, chemical fertilizer, nitrification, ammonia oxidizing bacteria, molecular docking analysis

## Abstract

Soil nitrogen (N) plays a critical role in plant nutrition and is regulated by the process of nitrification. Biochar can enhance soil N concentration and is hypothesized to influence nitrification, particularly when applied in combination with chemical fertilizers. Although, several studies have documented the effects of biochar and fertilizer combinations on nitrifying microbial communities, the underlying mechanisms remain poorly understood. In this study, the abundances of ammonia-oxidizing archaea (AOA) in the BT3 (15 t/ha) and BT5 (40 t/ha) treatments, and ammonia-oxidizing bacteria (AOB) in the BT4 (20 t/ha) treatment, were significantly higher than in fertilizer-only treatments. In contrast, nitrite-oxidizing bacteria (NOB) in the BT2 (5 t/ha) treatment exhibited the most significant difference (*p* < 0.05). Compared to the control (BT1), the relative abundances of AOB (Nitrosomonas; 51.74%) and NOB (Nitrolancea; 62.26%) increased significantly (*p* < 0.05) with higher application rates of biochar fertilizer concentration. Metabolic profiling and molecular docking simulations precisely demonstrated that 2,2-diethylacetamide (DEA), a compound structurally analogous to oxalate, interacts with the active site of pyruvate kinase, thereby affecting the glycolysis pathway. A subsequent potting experiment confirmed that DEA treatment increased pyruvate kinase gene expression (from 1.37 to 11.03; *p* < 0.05), soil pyruvic acid concentration (from 1.73 to 21.65; *p* < 0.05), and nitrifier abundance (from 0.20% to 0.56%; *p* < 0.05). Furthermore, soil copper (Cu; *R*^2^ = 0.21, *p* < 0.05), molybdenum (Mo; *R*^2^ = 0.53, p < 0.05), zinc (Zn; *R*^2^ = 0.37, *p* < 0.05), and total organic carbon (TOC; *R*^2^ = 0.24, *p* < 0.05) content were negatively correlated with nitrifier abundance as biochar-fertilizer application increased. This indicates that DEA derived from biochar, in conjunction with soil elemental composition, collectively influences nitrifying communities. The relationships between biochar-fertilizer application and nitrifier communities established in this study will inform precise application strategies to enhance nitrogen use efficiency in crops.

## 1 Introduction

Owing to agricultural development, a significant amount of N fertilizer is used in the agricultural industry to supply plants with nutrients (Jia et al., 2021). Nitrogen from fertilizers enters the soil and participates in the N cycle. The nitrification reaction is a crucial process in the soil N cycle. The nitration reaction is a two-phase process that includes ammonia oxidation and nitrite oxidation (Gruber and Galloway, 2008). In the ammonia oxidation process, N-${\text{NH}}_{4}^{+}$ is converted to ${\text{NO}}_{2}^{-}$ under the influence of ammonia-oxidizing bacteria (AOB) and archaea (AOA). ${\text{NO}}_{2}^{-}$ is then converted to ${\text{NO}}_{3}^{-}$ by nitrite-oxidizing bacteria (NOB) in the nitrite oxidation process (Jia and Conrad, 2009; Offre et al., 2009). Previous studies have shown that AOA and AOB are influenced by the complex soil environment (Liu et al., 2018), such as the soil pH, ammonia substrate, and organic molecules (He et al., 2012; Prosser and Nicol, 2012).

Biochar is a highly aromatic, carbon-rich solid material produced through the pyrolysis of biomass from agricultural and forestry residues under oxygen-limited or anaerobic conditions, typically at temperatures ranging from 350 to 700 °C. It exhibits high specific surface area, abundant porous structure, and stable chemical properties, which collectively contribute to its significant potential in soil amendment and heavy metal contamination remediation. Biochar enhances overall soil quality through multiple mechanisms, including regulation of soil cation exchange capacity (CEC), improvement of soil porosity and aeration, neutralization of acidity, and modulation of microbial community structure. Owing to its excellent adsorption capacity, biochar serves as an effective slow-release carrier in the formulation of biochar-based fertilizers. It facilitates the gradual release of nutrients while continuously improving soil physicochemical properties. By promoting the proliferation of beneficial microorganisms and suppressing the abundance of pathogens (such as reducing the proportion of harmful microbial groups like Fusarium) it effectively mitigates soil-borne diseases and continuous cropping obstacles. The biochar component within biochar-based fertilizers further regulates functional microbial communities involved in the biogeochemical cycling of elements such as carbon, nitrogen, and phosphorus. For instance, it enhances the activity of nitrogen-fixing and phosphate-soliloquizing bacteria, thereby improving the activation and transformation efficiency of key soil elements. This process increases nutrient availability and crop uptake efficiency. The activated nutrients can be re-adsorbed and retained by biochar, dynamically replenishing the nutrient pool depleted by plant uptake. Consequently, it significantly enhances soil nutrient cycling capacity while reducing issues such as soil compaction and non-point source pollution caused by excessive fertilizer application. In the ecological remediation of degraded soils, biochar-based fertilizers help modulate microbial communities by increasing the abundance of beneficial bacteria and reducing the competitiveness of pathogens. They inhibit the accumulation of autotoxic substances, and the organic small molecules on the surface of biochar can stimulate plants and microorganisms to secrete antimicrobial metabolites. This systemic resistance effectively alleviates continuous cropping obstacles. In summary, as a green, economical, and environmentally friendly functional material, biochar holds broad application prospects in promoting sustainable agricultural development and soil ecological restoration.

Many studies report that nitrification is generally a negative process for agriculture, such as N_2_O from nitrification processing. The N_2_O is one of the greenhouse gases. However, the genes in plant roots could recruit microbiota which have functions related to the nitrogen cycle in order to improving nitrogen utilization (Zhang et al., 2019). On the other hands, in our previous laboratory research, the different types of biochar have a positive effect on reducing N_2_O, although biochar have been promoted the related microbial gene express of nitrogen cycle (Li et al., 2021). Several studies have reported the effects of biochar organic compounds on soil microorganisms (Yang et al., 2021, 2015, 2019; Yuan et al., 2017).

Based on our previous research (Yang et al., 2015, 2019), we hypothesize that long-term biochar combined with chemical fertilizer treatment may lead to changes in the abundance and community structure of nitrifiers. The shifts may be derived from the organic compounds of biochar following long-term biochar fertilizer application. Therefore, this study focused on the effects on the bacterial community under different biochar combined with chemical fertilizer treatments, related metabolic substances in the soil, and biochar organic compounds. The main objectives of our experiment were to (1) investigate the abundance and communities of nitrifying microorganisms under biochar fertilizer treatment, (2) illustrate that the changes in the nitrifying microorganisms originate from organic compounds of biochar or the physical and chemical properties of the soil under biochar combined with chemical fertilizer treatment, and (3) understand the mechanism of the effect of special organic compounds from biochar on nitrifying microorganisms.

## 2 Materials and methods

### 2.1 Biochar and soil sampling

The tobacco stalk used in our experiment were obtained from Guizhou Tobacco Company in Bijie Company from China. The tobacco stalk were washed in ultrapure water for 5 min in order to removing the dust and naturally drying. And then, Biochar of tobacco stalk was prepared at a pyrolysis temperature of 400 °C at a heating rate of 3 °C/min. The final temperature was maintained for 2 h. Finally, biochar was cooled in natural environment.

The soil used in this experiment was collected from Qianxi Science and Technology Park, Guizhou Province (27 °1′N, 106 °1′E), which underwent long-term biochar fertilizer application from 2015 to 2018 for tobacco planting. Biochar combined with chemical fertilizer was applied as follow: BF, biochar (18%) with 8% nitrogen, 10% P_2_O_5_ and 22% K_2_O. The proportion of N:P:K is suitable for local fertilization practice in tobacco production. The BF treatments and non-biochar fertilizer treatments were arranged in a single-factor, five-level, three repeated random block design. The total applied BF concentrations were approximately 0 t/hm^2^ (BT1), 5 t/hm^2^ (BT2), 15 t/hm^2^ (BT3), 20 t/hm^2^ (BT4), and 40 t/hm^2^ (BT5). The biochar samples were collected from a depth of 0–5 cm at six random locations for each site and mixed as one soil sample for each replicate. All the soil samples were stored in an ultra-low temperature freezer (U725-86; Eppendorf, Germany). DNA extraction for molecular analysis and high-throughput sequencing was then performed.

### 2.2 Biochar compound analysis

Biochar contains polar and non-polar organic compounds that can affect the microbial community structure (Xiao et al., 2018). Therefore, based on the principle that compounds dissolve in materials with similar structures, different types of compounds (1.5 g) were extracted from the biochar with 100 mL of polar (methanol, ethanol, acetonitrile, chloroform, ethyl acetate, and dichloromethane) and non-polar (heptane and hexane) organic solvents. Gas chromatography-mass spectrometry (GC/MS) (7890A GC and 240-MS; Agilent, USA) was used to analyze the compounds in this study. A VF-5MS capillary column (30 m × 0.25 mm; Agilent, USA) was used for chromatographic separation. VF-5MS was not effective in the analysis of polar compounds. The polar organic solvent extracts (100 μL) were evaporated to dryness under nitrogen using a Termovap sample concentrator (MGS-200; Taitec, Japan). Then, 50 μL of methoxyamine hydrochloride in pyridine (20 mg/mL) was added as the derivatizing agent for derivatization. Hundred microliter of N,O-*bis*-(trimethylsilyl) trifluoroacetamide was added to the mixture for derivatization to identify the polar compounds using VF-5MS (Yang et al., 2019).

The extracted liquors were analyzed using GC/MS. The injection temperature was set at 280 °C with helium (99.999%) as the carrier gas (flow rate of 1 mL/min). The oven temperature was maintained at 40 °C for 1 min and then increased to 295 °C for 2 min. EI mode at 70 eV was programmed and scanned from 50 MHz to 650 MHz. Three replicates were used.

The total ion flow diagram obtained from GC/MS was analyzed using Wiley 6.0 (Wiley, New York, NY, USA), the Mass Spectral Library (Version 2.0, National Institute of Standards and Technology, NIST/EPA/NIH, USA), the Mass Bank, and the mass fragmentation pattern. The relative amount of each compound was determined by analyzing the total integrated area (1,000) per sample. Compounds with >80% probability in the library search program were identified as likely hits.

### 2.3 Physicochemical properties of biochar and soil

The biochar (1:10 w:w) and the soil (1:2.5 w:w) was prepared in ultrapure water and analyzed using a pH meter (S475-uMix; Mettler Toledo Company, Shanghai, China) for biochar and soil pH, respectively. Carbon and N analyses of the soil and biochar were performed using an elemental analyzer (vario MACRO cube; Elementar, Germany). The biochar and soil were digested using a microwave digestion system (ETHOS A; Milestone, Italy) to determine the elemental composition (Pathy et al., 2020). The digested liquid was then analyzed using ICP-MS (PerkinElmer NexION 350; PerkinElmer AG, USA). Then, 0.01 g of biochar and soil and 0.3 g of KBr were weighed using a ten-thousandth balance (EX225ZH/AD; Ohaus, USA). The mixture was compressed to wafers for Fourier transform infrared spectroscopy (FTIR; TENSOR27; Bruker, Germany) analysis to determine the functional groups of the biochar.

### 2.4 DNA extraction and qPCR analysis

DNA was isolated from the soil (0.5 g) using soil nucleic acid reagent (Takara, Dalian, China) following the manufacturer's instructions. The DNA quality and concentration were tested by gel electrophoresis and a NanoDrop spectrophotometer (NanoDrop Technologies, Wilmington, DE, USA). The integrity and uniformity of the DNA in each sample were confirmed by the amplification of Arch-amoAF/Arch-amoAR (forward primer: GATGGAGACTAGAACTGGAC; reverse primer: CTTACCAGCACCAAGGATGA) and amoA-1F/amoA-2R (ID: 825473; forward primer: GGAACGGCATCGGACCGAAG; reverse primer: AGCTCGATTCACGCTAGACGA).

An Illumina MiSeq sequencer (Illumina, San Diego, CA, USA) was used to analyze the 16S rRNA genes of the V3–V4 regions with the universal primers (515F: GTGCCAGCM GCCGCGG and 907R: CCGTCAATTCMTTTRAGTTT). MiSeq sequencing was performed by Novogene Co. (Beijing, China). All the polymerase chain reactions (PCRs) were conducted with 15 μL of Phusion^®^ High-Fidelity PCR Master Mix (New England Biolabs). Moreover, 0.2 μM of forward and reverse primers and approximately 10 ng of template DNA were used. Thermal cycling consisted of initial denaturation at 98 °C for 1 min, followed by 30 cycles of denaturation at 98 °C for 10 s, annealing at 50 °C for 30 s, elongation at 72 °C for 30 s, and final extension at 72 °C for 5 min. The PCR products were purified using a Qiagen Gel Extraction Kit (Qiagen, Germany) and quantified using a Nanodrop spectrophotometer (Thermo Scientific, Waltham, MA, USA).

The qPCR expression analysis was listed in S1.

### 2.5 MiSeq sequencing and phylogenetic analysis

Quality filtering on the raw tags was performed under specific filtering conditions to obtain high-quality clean tags (Bokulich et al., 2013) according to the QIIME (V1.9.1; http://qiime.org/scripts/split_libraries_fastq.html; Caporaso et al., 2010) quality-controlled process. A total of 422 530 high-quality 16S rRNA gene sequence reads were obtained. Operational taxonomic unit abundance information was normalized using a standard sequence number corresponding to the sample with the least sequences. Subsequent analysis of alpha and beta diversity was performed based on the normalized output data.

### 2.6 Metabonomics of soil analyzed

A Vanquish UHPLC system (Thermo Fisher) coupled with an Orbitrap Q Exactive HF-X mass spectrometer (Thermo Fisher) was used to analyze the metabonomics of soil and quantify the compounds. The operation procedure is shown in S1.

### 2.7 Pot experiment

In order to understand the DEA biological function, the pot experiments was used in our experiment. Pot soils from Weining in China. The experiment was conducted at Zunyi Normal College, Guizhou Province, China (27 °13′15″ N, 106 °17′22″ E). The size of the pots used in this part of the experiment was 35 cm × 35 cm with 5 kg of soil. The soil used in the pot experiment had the following properties: pH 5.12, total carbon (TC) 3.2%, total nitrogen (TN) 0.34%, TC:TN (C:N) 9.42, available ${\text{NH}}_{4}^{+}$-N 2.55 mg/kg, ${\text{NO}}_{3}^{-}$-N 126.14 mg/kg, ${\text{PO}}_{4}^{3-}$-P 25.28 mg/kg, K 217.53 mg/kg, extractable S 0.04%, Mg 0.26%, Ca 0.32% and Al 12.3 mg/g (see below for the methods for soil analysis). According to the local fertilization practices in Weining, chemical fertilizer (N: 6.6 g, P_2_O_5_: 5.4 g and K_2_O: 14.2 g for every pot) was applied in our pot experiment to provide nutrients for tobacco growth. In each pot, one tobacco plant was planted. The pots included 100 g/kg DEA per pot, comparing non-DEA as control. Every treatment included three repetitions.

### 2.8 Statistical analysis

All the numerical data were analyzed using the statistical software SPSS (version 26.0) and Microsoft Excel (version 2016). The results of potential biochar significant effect on 16s RNA, ITS and *amoA* genes were calculated at the level of significance of *p* < 0.05 or < 0.01 using Tukey's *t*-test. ^*^ indicated *p* < 0.05, ^**^ showed *p* < 0.01. The Pearson correlation coefficient was employed to analyze the relationships between *amoA* genes abundance and elements of soil under biochar combined with chemical fertilizer application (or organic compounds from biochar).

However, the precise biochar substance that affects nitrifying communities is unknown. Therefore, the independent determinants of organic compounds from the GC/MS analysis results were screened using binary logistic regression analysis. Candidate variables with *p* < 0.2 in univariate analysis were included in the multivariable model. Statistical significance was defined as a two-sided *p*-value of < 0.05. Finally, the multivariable model results (*p* < 0.05) identified the organic compounds changing the nitrification microorganism composition.

### 2.9 Molecular docking analysis: blind docking simulation

Seventeen organic compounds identified by GC/MS were constructed using GaussView 6.0, and the structure was optimized based on B3LYP/6-311G^**^. The protein receptor pathway was downloaded from the Research Collaboratory for Structural molecular docking protein database (https://www.rcsb.org) to construct autodocking data. AutoDock Vina was used to dock all ligands (determined by GC/MS analysis of biochar) and protein receptors. The Vina scoring system was described by Trott and Olson (2010). The PyMOL software package was used to visualize docked conformations.

## 3 Results and discussion

### 3.1 Biochar and soil characterization

**Figure 1:**
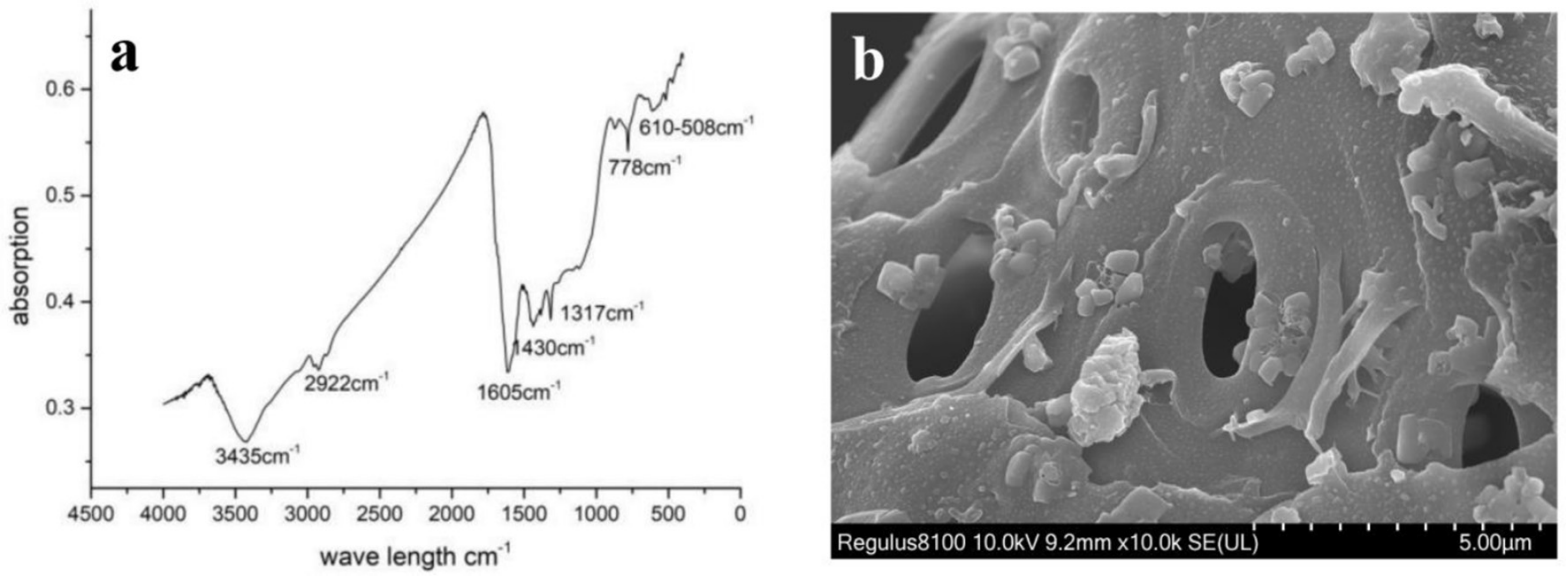
The result of Fourier transform infrared spectroscopy **(a)** and SEM **(b)** from biochar at 400 °C.

The FTIR analysis showed that biochar had very weak absorbance. Four groups of signals were detected at 3,435 cm^−1^ denoting OH, 2,922 cm^−1^ denoting C-C-H, 1,605 cm^−1^ denoting aromatic ring C=C bonds, and 1,317 cm^−1^ denoting C-H stretching (Figure 1a). The biochar yield from tobacco stems was 30.82%, with an ash content of 11.50%. The cell structure of the precursor tobacco stems is shown in the scanning electron microscopy images (Figure 1b). Many pores (11 μm) were identified in the biochar samples. The results indicated that the pores may promote microbial growth by providing a location for growth (Xiao et al., 2018). The previous studies reported that the structure of the microbial population in the soil was affected by the biochar pores, such as providing shelters for microorganisms. In our study, the surface area of the biochar, as determined by N_2_-BET, was 35 m^2^/g. The pores and BET did not affect the nitrifying community (*p* > 0.05) in our study. The total carbon (TC) (wt. %) and total nitrogen (TN) (wt. %) contents of biochar were 51.41 and 2.57, respectively. The pH of the extract was approximately 10.30. According to the ash content, the C:N ratio was approximately 20%. The rapidly available total organic carbon (TOC) and N contents were 4.4341 and 1.0300 mg/g, respectively. The biochar conductivity was 5,270 μs/cm. The chemical elements of biochar are presented in Table 1. Zn (113.38 mg/kg), Ca (49.22 mg/kg), Cu (33.79 mg/kg), and C (51.41%) were the main elements in the biochar. Minor elements played a role in regulating the growth process of microorganisms from soil. Therefore, the physical and chemical properties of biochar affected the soil characterization, which was also analyzed in this study.

**Table 1:** Physical and chemical properties of biochar.

**Physical and chemical properties of biochar**	**Concentration**
C_concent	51.41
N_concent	2.57
C:N	20.01
TOC	4.43
pH	10.30
EC	5270.67
Carbon production rate (%)	38.37
B (mg·kg^−1^)	1.10
Mn (mg·kg^−1^)	0.55
Cu (mg·kg^−1^)	33.79
Zn (mg·kg^−1^)	113.38
Mo (mg·kg^−1^)	1.31
Na (g·kg^−1^)	5.32
Mg (g·kg^−1^)	6.52
P (g·kg^−1^)	9.35
Ca (g·kg^−1^)	49.22
Fe (g·kg^−1^)	4.10

As shown in Tables 2, 3, the TOC (mg/L) and TC (mg/L) concentrations in soil increased and decreased with biochar application intensity increased, and TOC (mg/L) reached a peak in treatment BT2 (5 t/hm^2^). Contrarily, TN (mg/L) showed a trend of first decreasing and then increasing and reached a peak in treatment BT5 (40 t/hm^2^). The TN (mg/L) concentration in treatment BT3 (15 t/hm^2^) was significantly lower than that in the other treatments; however, the TC (mg/L) concentration in treatment BT3 (15 t/hm^2^) was significantly higher than that in the other treatments. The C, N, P, Fe, S, Mn, Cu, Zn, and Mo contents in the soil increased after long-term biochar application. Among them, the contents of K, Fe, Mn, Zn, and Mo in treatment BT4 (20 t/hm^2^) were significantly higher than those in treatment BT1 (0 t/hm^2^) (12.2%, 27.9%, 30.6%, 58.2%, and 68.5%, respectively), whereas the contents of Na, Mg, and Ca in the soil decreased. The contents of Na, Mg, and Ca in treatment BT1 (0 t/hm^2^) were significantly higher than those in treatment BT5 (40 t/hm^2^) (45.7%, 8.1%, and 32.6%, respectively). The results indicated that the microbial living environment in the soil changed. However, the changed environment was mainly derived from the effects of biochar. Therefore, the microbial composition was indirectly affected by the biochar content.

**Table 2:** Physical and chemical properties of soil.

**Treatment**	**PH**	**CE (μs/cm)**	**TOC (mg/L)**	**IC (mg/L)**	**TC (mg/L)**	**TN (mg/L)**	**N (%)**	**C (%)**
BT1	6.36bc	2049.67a	50.67ab	0.80b	215.79ab	1.27ab	0.27a	1.95b
BT2	6.03cd	2211.67a	62.43a	0.58b	306.59a	1.20ab	0.26a	1.72d
BT3	6.81a	1908.00a	48.22ab	2.92a	69.72c	1.12b	0.26a	1.81cd
BT4	6.46ab	2084.67a	45.29b	1.35b	128.97bc	1.30ab	0.26a	1.90bc
BT5	5.73d	1883.00a	46.68ab	0.54b	322.00a	1.40a	0.27a	2.11a

CE stands for conductivity; TOC stands for total organic carbon; IC stands for inorganic carbon; TC stands for total carbon; TN stands for total nitrogen. The total applied biochar concentrations were approximately 0 t/hm^2^(BT1), 5 t/hm^2^(BT2), 15 t/hm^2^(BT3), 20 t/hm^2^(BT4), and 40 t/hm^2^(BT5). Different lowercase letters within the same column indicate significant difference among different treatments at the level of *p* < 0.05.

**Table 3:** Elements concentration of soil.

**Treatment**	**Na (g/kg)**	**Mg (g/kg)**	**P (g/kg)**	**K (g/kg)**	**Ca (g/kg)**	**Fe (g/kg)**	**B (mg/kg)**	**S (mg/kg)**	**Mn (mg/kg)**	**Cu (mg/kg)**	**Zn (mg/kg)**	**Mo (mg/kg)**
BT1	0.51a	3.88a	1.35a	11.16b	1.79a	50.49b	110.23a	317.69a	796.98b	32.94b	31.79b	0.92b
BT2	0.54a	3.62abc	1.94a	10.69b	1.70ab	53.96b	108.54a	371.79a	937.68ab	42.96ab	46.53a	1.29ab
BT3	0.50a	3.72ab	1.29a	11.67ab	1.88a	53.73b	126.84a	321.24a	807.84b	51.15a	52.95a	1.58a
BT4	0.43ab	3.34c	1.59a	12.52a	1.57ab	64.56a	107.46a	359.53a	1040.58a	42.37ab	50.28a	1.55a
BT5	0.35b	3.59bc	2.09a	10.83b	1.35b	52.44b	126.97a	400.93a	797.82b	34.75ab	40.30ab	1.35a

Lowercase letters (a, b, c) are significantly different (*p* < 0.05, one-way ANOVA followed by Tukey's post hoc test).

### 3.2 Responses of nitrifying communities to biochar treatment

**Figure 2:**
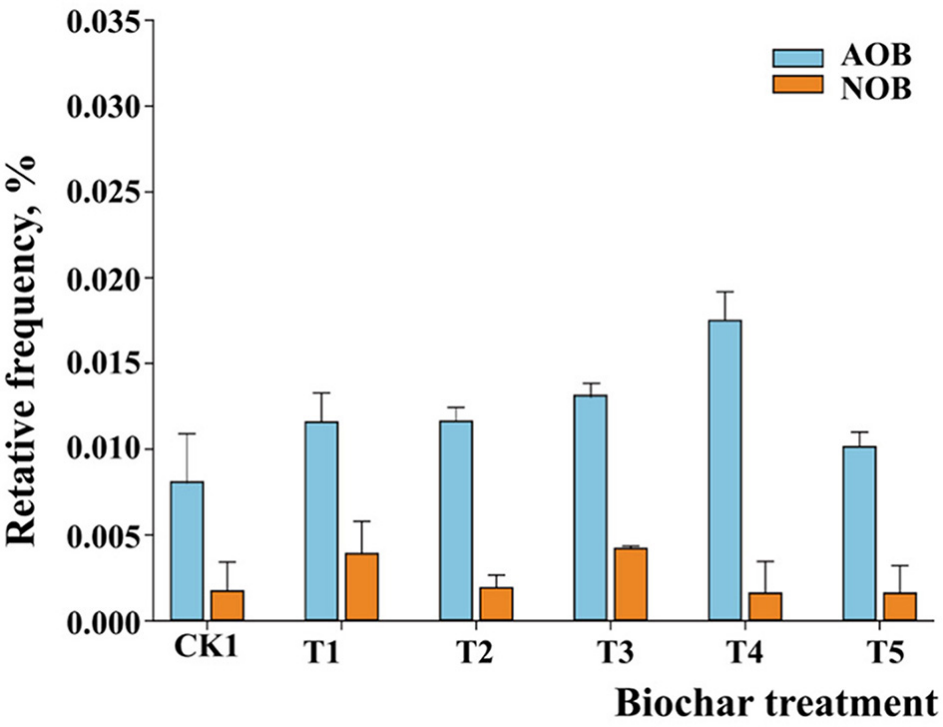
Changes in the copy numbers of ammonia-oxidizing bacteria (AOB) and nitrite-oxidizing bacteria (NOB) 0 t/hm2(BT1), 5 t/hm2(BT2), 15 t/hm2(BT3), 20 t/hm2(BT4), and 40 t/hm2(BT5).

High-throughput sequencing of 16S rRNA is a powerful tool for analyzing changes in the relative abundance of the microbial population. We obtained 950,680 high-quality sequences from 15 samples. A sample average 63,378 sequence. The VSEARCH was employed to analyze the high-quality sequence to removing chimeric and organelle sequence, cut-off threshold was set in 1.5. The 5,450 good sequence were obtained from denosise for predicting biological sequences. The RDP and SILVA database were employed to remove plastid and non-bacteria with cut of 0.1 which obtained 5,130 sequences. Normalize by subsample with depth 10,000 and seed 1 were conducted by Vegan package. In this part, AOB and NOB were enriched from 0.150% and 0.012% in the control (BT1) to 0.170% (BT4) and 0.020% (BT2) (*p* < 0.05), respectively. As shown in Figure 2, as the BF concentration increased, the abundance of AOB first decreased (BT2) and then increased (BT4). In treatment BT5 (40 t/hm^2^), the abundance of AOB decreased again. This change might have been due to the biochar pH (10.3). When biochar was added to the soil, the pH of the soil changed from 6.4 to 8.2 and became alkaline (*p* < 0.05). Thus, AOB became the dominant microorganisms (Chu et al., 2007; Yang et al., 2021; Shen et al., 2008; Wang et al., 2013; Wu et al., 2011). AOA might be inhibited in an alkaline environment. AOA were also not detected in our experiment using MiSeq sequencing. However, the abundance of NOB first increased (BT2) and then decreased (BT3). Finally, the abundance of NOB was stable (0.09%; BT4 and BT5). The results indicated that appropriate concentrations of BF could promote AOB and NOB growth. However, nitrifying communities were inhibited by high BF concentrations. Therefore, the 20 t/hm^2^ BF treatment (BT4) was suitable for ammonia oxidation (promoting AOB growth) and the 5 t/hm^2^ BF treatment (BT2) was suitable for nitrite oxidation (promoting NOB growth) in the field experiment.

**Figure 3:**
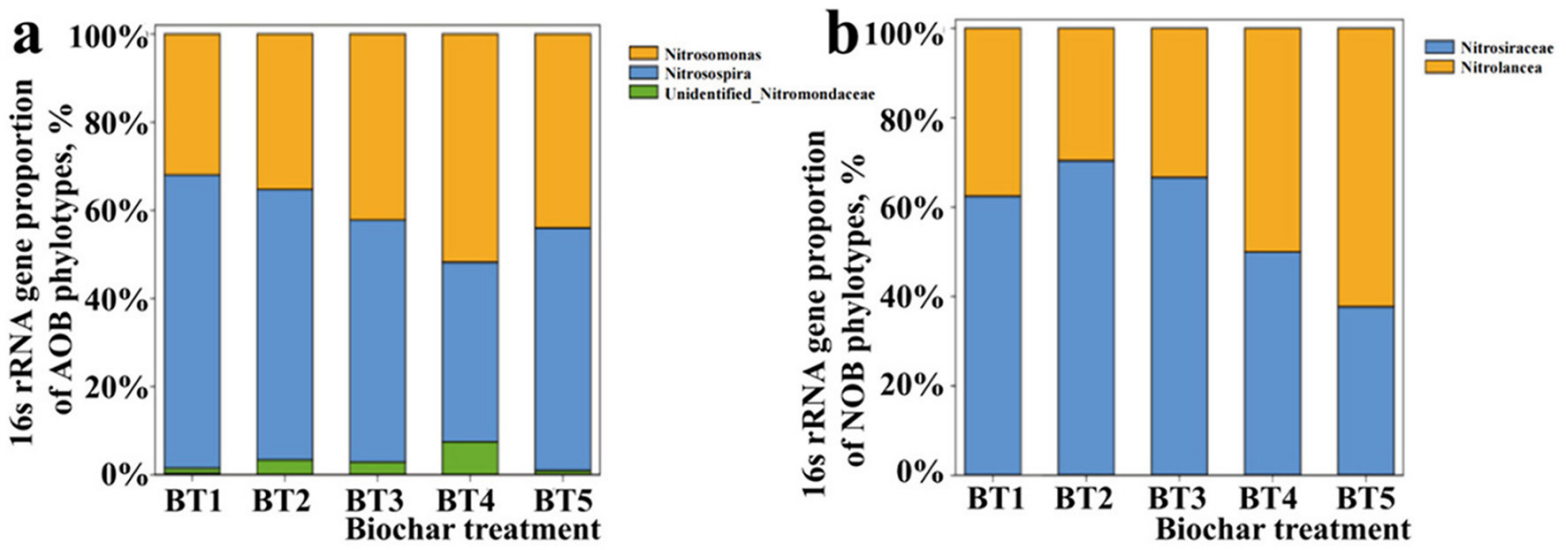
Proportional changes of nitrifying phylotypes of **(a)** ammonia-oxidizing bacteria and **(b)** nitrite-oxidizing bacteria in response to long-term biochar application.

The change in microbial communities was affected by the different biochar fertilizer concentrations. Regarding AOB, *Nitrosospira* was the dominant bacteria (66.51%; *p* < 0.05) in treatment BT1 (CK) (Figure 3a). However, as the BF concentration increased, the *Nitrosospira* community decreased. Unidentified_*Nitrosomonadaceae* (20 t/hm^2^ BF treatment) became the dominant bacteria (51.74%, *p* < 0.05). *Nitrosomonas* and Unidentified_*Nitrosomonadaceae* communities were promoted by the increase in BF concentrations. The two communities were inhibited by the 40 t/hm^2^ BF treatment. *Nitrosospira* was the dominant bacterium. However, among NOB, as the BF treatment concentration increased (Figure 3b), *Nitrolancea* (62.26%; *p* < 0.05) became the dominant bacteria and reached its peak in treatment BT5 (40 t/hm^2^). However, unidentified_*Nitrospiraceae* was inhibited by treatments BT3–BT5. These results prove that BF can affect the microorganism composition of nitrification.

**Figure 4:**
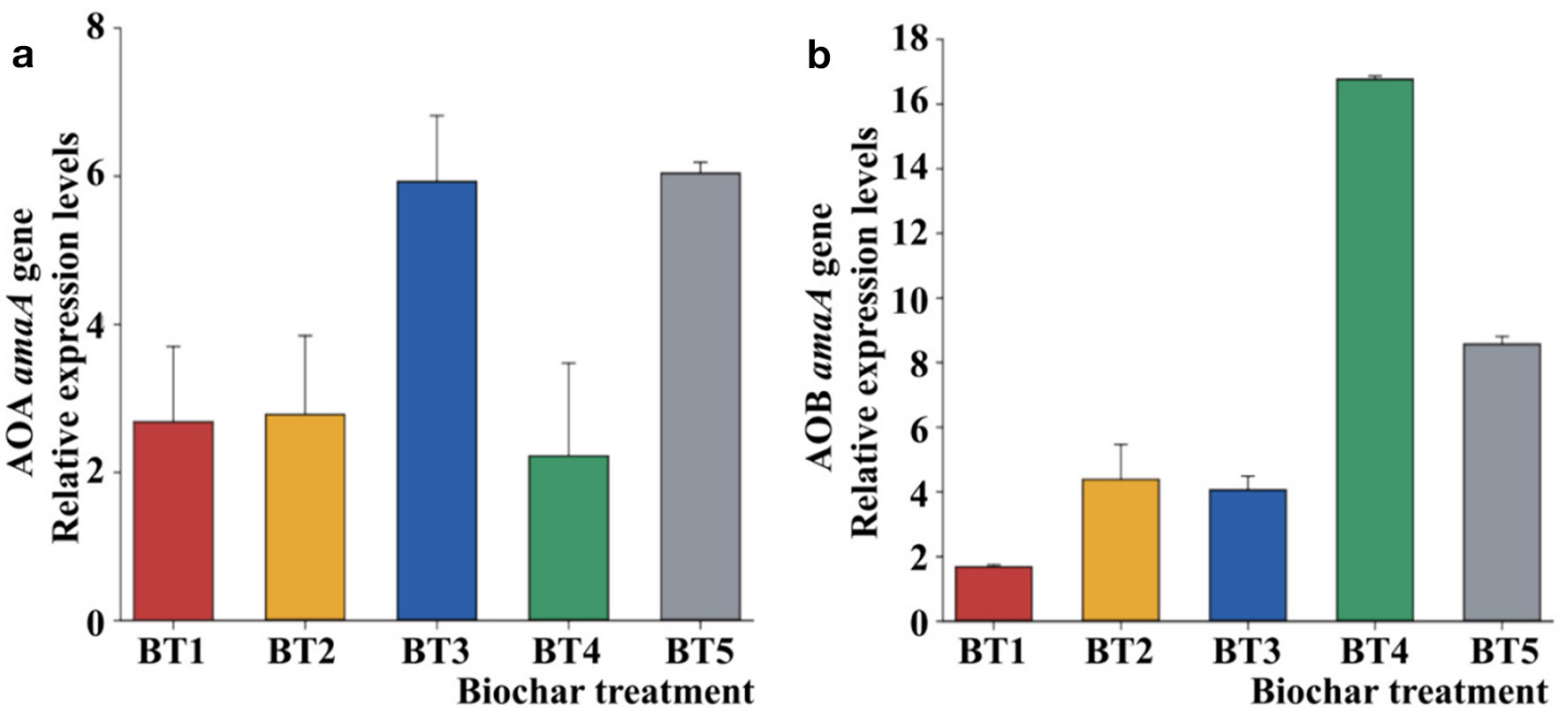
Quantitative RT-PCR analysis of archaeal **(a)** and bacterial **(b)** amoA genes in different soils. Error bars represent the standard errors of the triplicate microcosms. The different letters above the columns indicate a significant difference (*P* < 0.05) based on the analysis of variance.

AOA clusters were not observed by high-throughput sequencing. Therefore, owing to the ability of BF to promote the microbial community, the abundance of archaeal and bacterial amoA genes in soil samples was analyzed by quantitative real-time PCR to determine the mechanism by which BF changes the nitrifying microorganisms in soil. At higher BF concentrations, the gene expression of AOA and AOB in soil samples was promoted (Figures 4a, b). However, the AOA genes in BT4 were suppressed upon treatment with 20 t/hm^2^ of BF (Figure 4a). The results showed that a high concentration of biochar was beneficial to the AOA archaeal community.

The AOB gene expression (from 0.81 × 10^3^ to 16.3 × 10^3^) gradually increased as the biochar concentration increased (Figure 4b). However, the AOB in BT5 (40 t/hm^2^) were inhibited by the high biochar concentration. This result is consistent with that of the growth of rice seedlings, which was inhibited by biochar liquor (5% biochar liquor treatment; Yang et al., 2019). However, in the field trial, the biochar dosage was higher than that of the water culture (5% biochar treatment), and the same trend was observed. The results suggested that the biochar compounds could have directly affected the plants or bacteria in the biochar water culture experiment (Yang et al., 2019, 2015). Therefore, different biochar concentrations were used for the water culture. Nevertheless, the biochar concentration in the field trial was equal to that diluted by the soil. The N-rich and organic compounds of BF affected the abundance of soil nitrifying microorganisms.

### 3.3 Compositions of compounds from biochar extracts

The high-throughput sequencing and qPCR results indicated that the nitrification microorganisms were changed by the BF. The mechanism by which biochar changes the composition of microorganisms is unknown. Lehmann reported that there are more abundant molecules in the pyrolysis process at 400 °C than at other pyrolysis temperatures (Lehmann and Joseph, 2009). In our preliminary test, the growth of plants and microorganisms in the soil was promoted by the organic compounds of biochar at a pyrolysis temperature of 400 °C (Yang et al., 2019, 2021). Biochar pyrolysis at 400 °C was used in the field experiment in this study. Therefore, we considered that the organic compounds of biochar were the key factors changing the microorganisms in nitrification. Therefore, GC/MS was used to analyze the constituents of the compounds in the biochar extracts. The results indicated that 17 organic compounds were detected in the biochar extracts (Table 4). Various functional groups were identified in the 17 compounds, including alcoholic hydroxyls, esters, carboxylic acids, alkyls, ether bonds, aldehydes, carbonyls, and phenolic hydroxyls. The special functional groups of organic compounds interact with the receptor proteins of N-related bacteria, which promotes changes in the soil microbial community (Yang et al., 2015).

**Table 4:** The compounds from biochar extracts identified by GC/MS.

**Num**	**Name**	**Formula**	**Rt (min)**
1	Pentanamide, 2-(dimethylamino)−4-methyl-N-[2-methyl-1-[[3,3a,11,12,13,14,15,15a-octahydro-12,15-dioxo-13-(phenylmethyl)−5,8-ethenopyrrolo[3,2-b][1,5,8]oxadiazacyclotetradecin-1(2 H)-yl]carbonyl]butyl]-Pyridine	C_36_H_49_N_5_O_5_	2.260
2	Pyridine	C_5_H_5_N	2.755
3	6-(Methylthio)hexa-1,5-dien-3-ol	C_7_H_12_OS	4.523
4	Formamide, N,N-diethyl-	C_5_H_11_NO	5.759
5	Ethanamine, N-pentylidene-	C_7_H_15_N	5.925
6	trans-2,4-Dimethylthiane, S,S-dioxide	C_7_H_14_O_2_S	6.666
7	Acetamide, N,N-diethyl-	C_6_H_13_NO	7.022
8	2,2-Diethylacetamide	C_6_H_13_NO	7.158
9	Cyclopentanone, 2-(1-methylpropyl)-	C_9_H_16_O	7.821
10	2-Acetyl-5-methylfuran	C_7_H_8_O_2_	16.096
11	Tritetracontane	C_43_H_88_	18.441
12	Pyrrolidin-2-one, 1-[1-(4-carbomethoxyphenyl)butan-1-ol-2-yl]-	C_15_H_12_C_l2_O_3_	2.225
13	(1 R,2 R,4 S)−2-(6-Chloropyridin-3-yl)−7-azabicyclo[2.2.1]heptane	C_11_H_13_ClN_2_	2.076
14	Acetamide, N,N-diethyl-	C_4_H_9_N	4.892
15	4,6-Dimethyl-2-thioxo-1,2-dihydro-3-pyridinecarbonitrile tbdms	C_14_H_22_N_2_SSi	2.101
16	1-Oxa-4-azaspiro[4.5]decan-4-oxyl, 3,3-dimethyl-8-oxo-	C_10_H_16_NO_3_	5.757
17	Cyclopentanone, 2-(1-methylpropyl)-	C_9_H_16_O	4.538

### 3.4 Docking analysis

**Figure 5:**
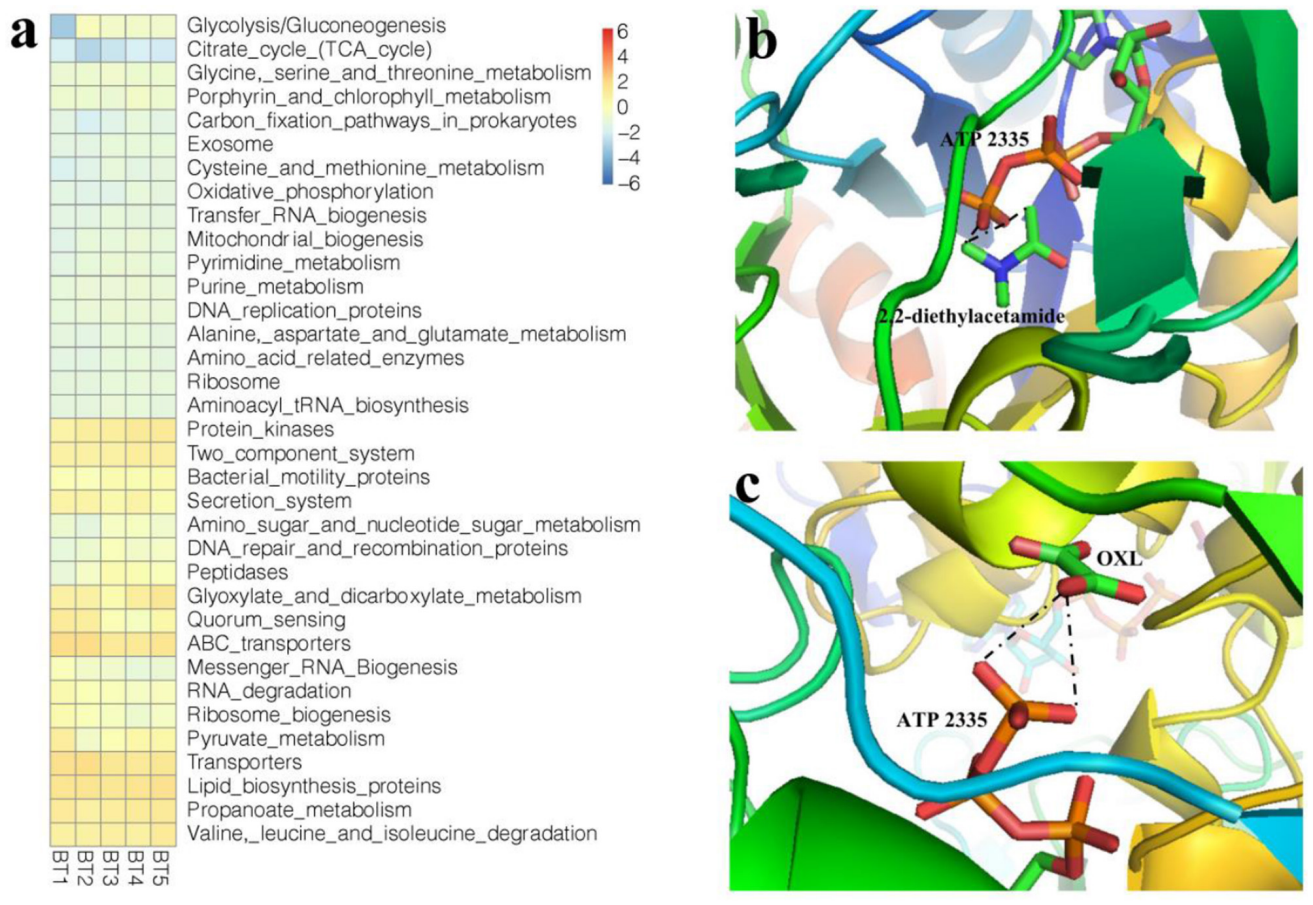
The function analysis from metabonomic pathway **(a)**. Molecular structure illustrating the interaction among 2,2-diethylacetamide **(b)**, OXL **(c)** and pyruvate kinase.

The results presented in Section 3.3 indicated that there were relationships between the four organic compounds from biochar and the nitrification microorganism composition. Therefore, we considered that these four organic compounds affected the nitrifying communities in the nitration reaction. The biological functions were analyzed using the metabonomics data from the soil. The results indicated that the metabolic pathways of the soil microbial population were changed (Figure 5a). The metabolism of soil microorganisms, such as glycolysis, was promoted by BF treatment (*p* < 0.05). The function of most pathways is related to energy metabolism. Thus, BF enhanced the microbial activity. However, this mechanism of BF is unknown. Therefore, the interactions between the biochar molecules (GC/MS result) and target receptors were explored by performing a docking simulation to understand which biochar molecules affected the function of soil microorganisms. DEA deeply entered the active pocket of *Oryctolagus cuniculus*, which is a favorable binding site. The interactions among DEA from biochar, the oxalate ion (OXL) from the glycolysis pathway, and pyruvate kinase are shown in Figures 5b, c. The H bond was mainly a chemical combination of DEA and the functional domain of pyruvate kinase. When DEA entered the functional domain of pyruvate kinase, the molecule could form H with the ATP-2335 from another ligand of pyruvate kinase (Figure 5b). However, OXL could interact with ATP via H bonding (Figure 5c). The results showed that there was a different position of the H bond between DEA and OXL, but the acting functional domain was the same. Previous studies have indicated that OXL plays an important regulatory role in the glycolysis metabolic pathway (Li et al., 2019). However, according to the soil metabonomics, the related organic compounds of the glycolysis metabolic pathway and the nitrifying communities interrelated with biochar treatment increased (*p* < 0.05; *r* = 0.875). Therefore, we considered that the nitrifying communities were promoted by DEA via promoting pyruvate kinase gene expression in the theoretical analysis. Other organic compounds of biochar cannot enter any active domain of protein from the pathway shown in Figure 5a. The results of regressive analysis indicated that of the 17 organic compounds of biochar, 2-acetyl-5-methyfuran, pyridine, DEA, and N, N-diethyl-formamide (*p* < 0.05) could affect the nitrification microorganism abundance and composition.

### 3.5 Contrasting effects of biochar-derived organic compounds

**Figure 6:**
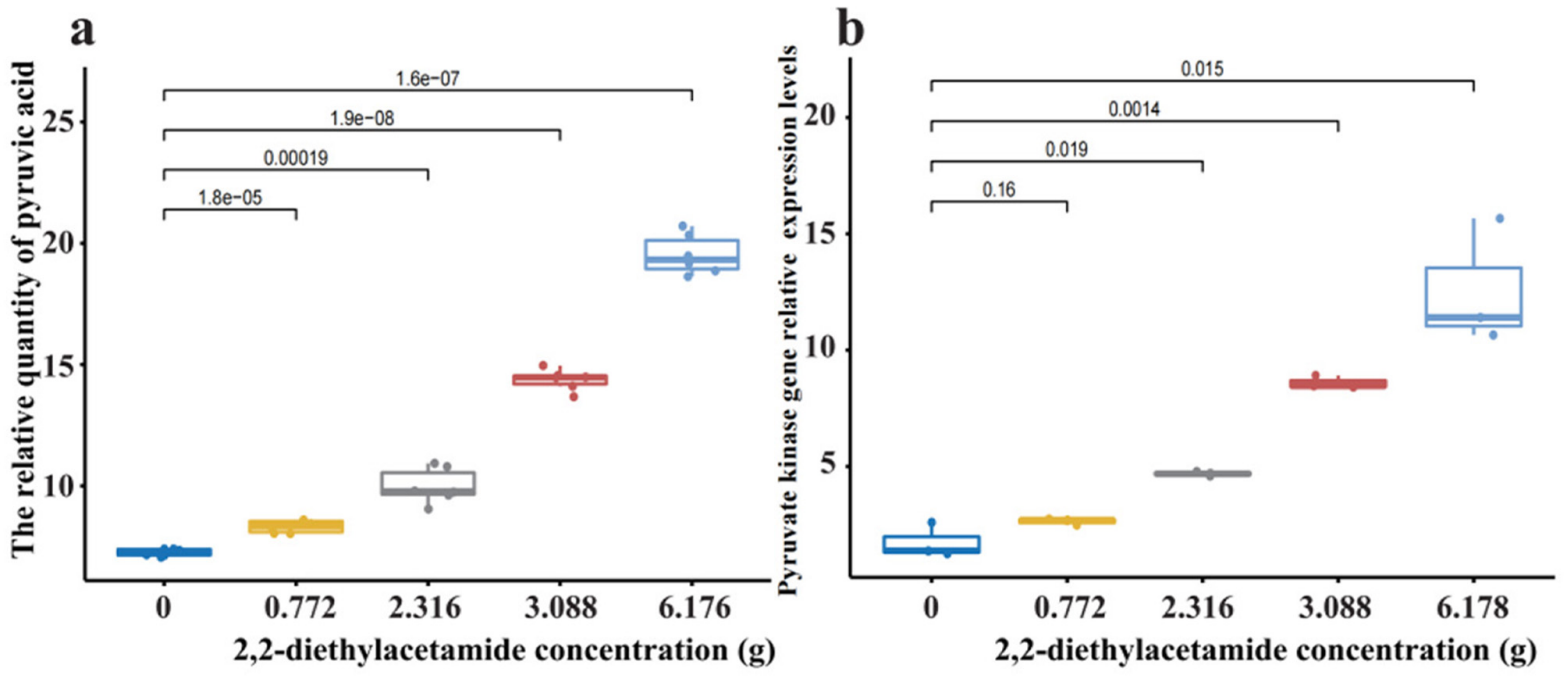
2,2-diethylacetamide **(a)** enhanced the pyruvic acid concentration and **(b)** promoted pyruvate kinase the gene expression.

**Figure 7:**
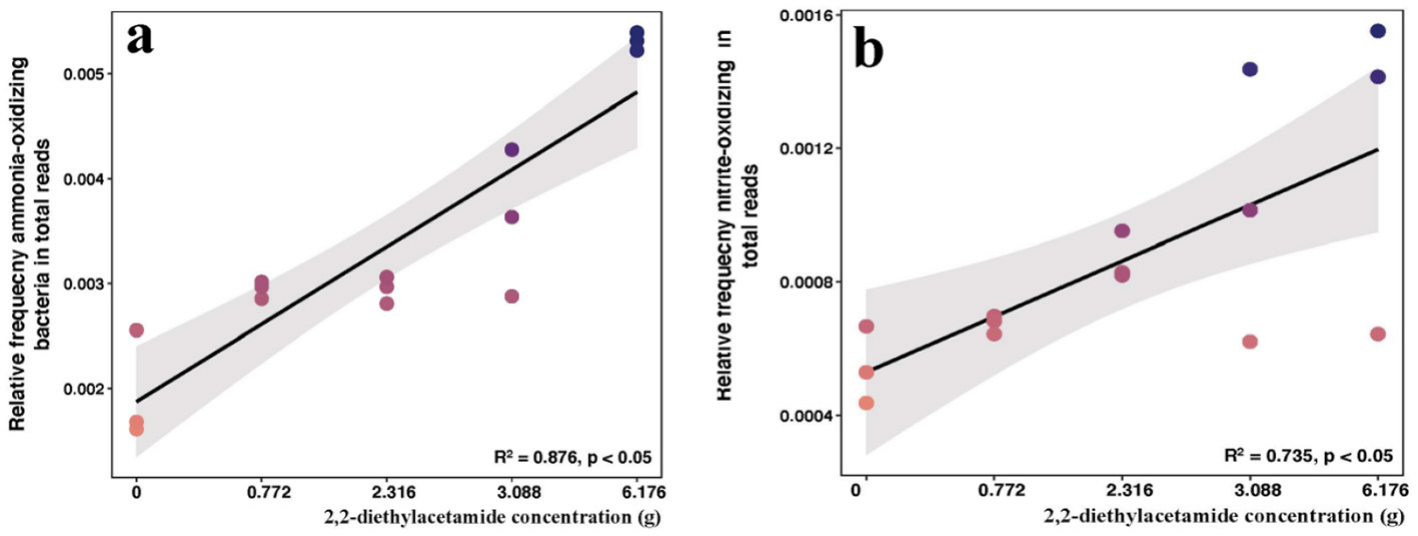
2,2-diethylacetamide concentration was positive correlation with the ammonia-oxidizing **(a)** and nitrite-oxidizing **(b)** bacteria with linear increase in pot experiment (*p* < 0.05).

Different concentrations of pure DEA were added to potting soil to confirm the biological function of DEA by theoretical analysis. The DEA concentration in the potting soil was based on a previous biochar treatment that contained DEA. The results indicated that high pyruvate kinase gene expression should promote the increase in the pyruvic acid concentration (*p* < 0.05; Figure 6a). Pyruvic acid is a metabolic substance derived from pyruvate kinase. Meanwhilie, pyruvate kinase gene expression was promoted by the increase in DEA (*p* < 0.05; Figure 6b). However, high pyruvate kinase gene expression should promote the increase in the pyruvic acid concentration. Pyruvic acid is a metabolic substance derived from pyruvate kinase. Pyruvic acid was measured in our experiment using UPLC-MS/MS. The peak area was used as the relative quantity of pyruvic acid (Figure 5a). The results indicated that pyruvic acid was also promoted in different DEA treatments (*p* < 0.05). There was a linear increase in the abundances of AOB and NOB (Figure 7). The tendency of AOB and NOB first increasing and then decreasing was not inhibited by the high DEA treatment (Figures 2, 3). DEA promoted the growth of nitrifying communities (*p* < 0.05).

**Figure 8:**
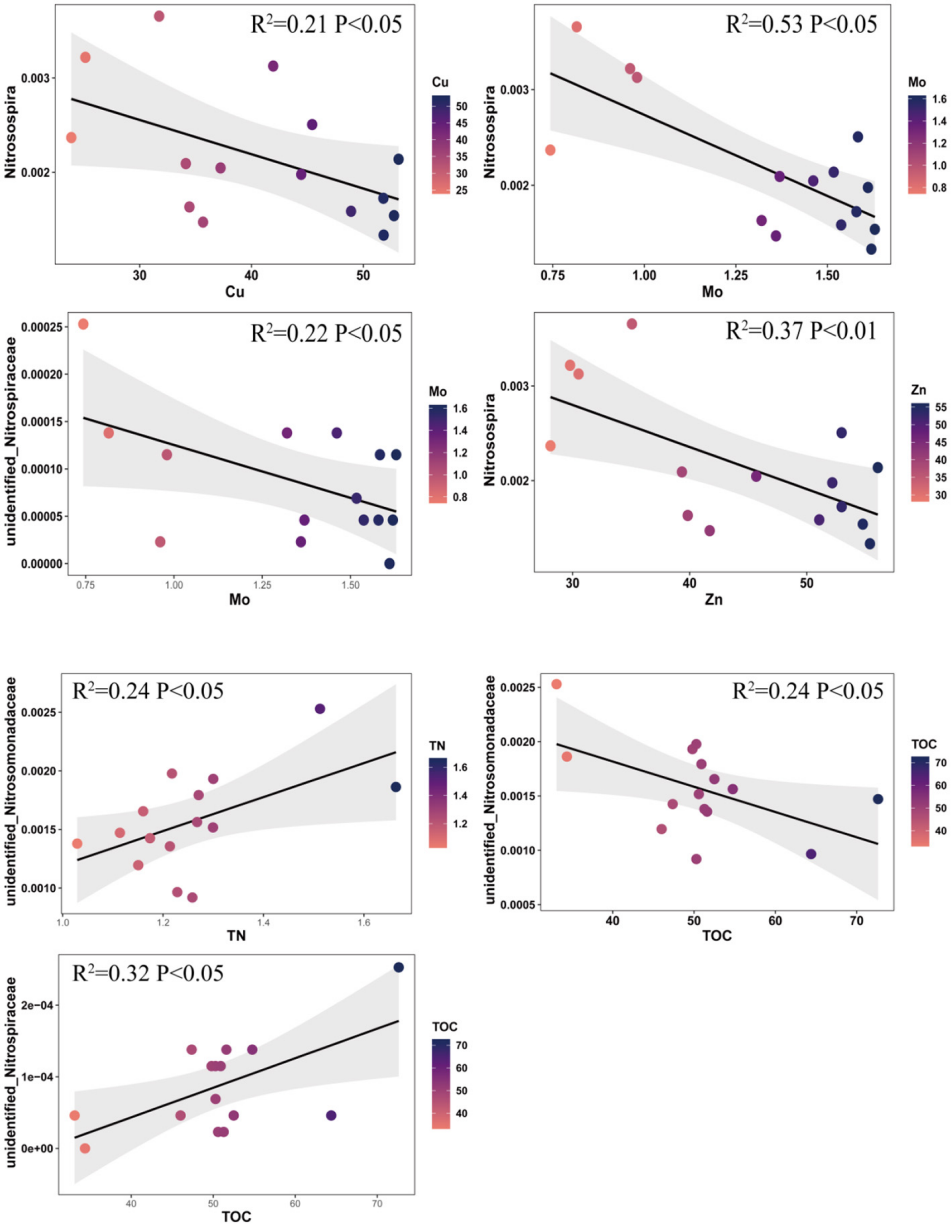
Correlation between elements of soil and nitrifying communities.

For our data between the nitrifying communities and the physicochemical properties of the soil (or biochar organic compound), there are linear relationship, Variance Inflation Factor (VIP) < 5, non-outlier. So, the relationship between the nitrifying communities and the physicochemical properties of the soil was analyzed to explain the growth of different nitrifying communities under DEA and BF (Figure 8). The results suggested that Cu (*R*^2^ = 0.21; *p* < 0.05), Mo (*R*^2^ = 0.53; *p* < 0.05), Zn (*R*^2^ = 0.37; *p* < 0.05), and TOC (*R*^2^ = 0.24; *p* < 0.05) were negatively correlated with *Nitrosospira*. Mo (*R*^2^ = 0.22; *p* < 0.05) showed a negative interaction with unidentified_*Nitrosomonadaceae*. However, there was a positive correlation between TN (*R*^2^ = 0.0.53; *p* < 0.05) and TOC (*R*^2^ = 0.32; *p* < 0.05) and unidentified_*Nitrosomonadaceae*. The physicochemical properties of the soil, including the pH and TC content, did not affect the nitrifying communities (*p* > 0.05). Therefore, we considered that DEA could promote the growth of nitrifying communities. However, various elements from BF inhibited the growth of nitrifying communities.

## 4 Conclusion

Tobacco plantation soil was selected to investigate how the abundance and community structure of nitrifiers changes upon long-term biochar application. BF application significantly increased the abundances of AOA and AOB in treatments BT3 (15 t/hm^2^), BT4 (20 t/hm^2^), and BT5 (40 t/hm^2^). Biochar treatment also changed the community structure of nitrifying populations, especially in treatments BT2 (NOB) and BT4 (AOB). However, the underlying mechanisms remain unclear. Functional analysis of metabonomics showed that BF can significantly affect the glycolysis pathway. Theoretical analysis of molecular docking also proved that the DEA from biochar played an important role in the glycolysis pathway. Potting experiments showed that pyruvate kinase, pyruvic acid, and nitrifier abundance were promoted by DEA treatment. However, the Mo, Cu, Zn, and TOC contents from BF were negatively correlated with nitrifying communities. Therefore, the DEA from biochar and the elemental composition of the soil together affected the nitrifying communities.

## Data Availability

The original contributions presented in the study are included in the article/Supplementary material, further inquiries can be directed to the corresponding authors.
