# Supplementary material for: Effects of long-term biochar application combined with chemical fertilization on the nitrifier community in tobacco plantation soil
Source: Front Microbiol. 2025 Dec 11;16:1720769. doi: 10.3389/fmicb.2025.1720769 (PMC12738806; doi:10.3389/fmicb.2025.1720769)
Supplement: Supplementary file 1 [file Data_Sheet_1.docx]

**Effects of long-term biochar-****fertilizer application to the soil of tobacco plantations on ammonia oxidizers and nitrite-oxidizing bacteria**

**Untargeted Metabolomics - Materials and Methods**

**1. Metabolites Extraction**

Tissues (100 mg) were individually grounded with liquid nitrogen and the homogenate was resuspended with prechilled 80% methanol and 0.1% formic acid by well vortexing. The samples were incubated on ice for 5 min and then were centrifuged at 15000 rpm, 4°C for 5 min. A some of supernatant was diluted to final concentration containing 60% methanol by LC-MS grade water. The samples were subsequently transferred to a fresh Eppendorf tube with 0.22 μm filter and then were centrifuged at 15000 *g*, 4°C for 10 min. Finally, the filtrate was injected into the LC-MS/MS system analysis.

**2. UHPLC-MS/MS Analysis**

LC-MS/MS analyses were performed using a Vanquish UHPLC system (Thermo Fisher) coupled with an Orbitrap Q Exactive HF-X mass spectrometer (Thermo Fisher). Samples were injected onto an Hyperil Gold column (100×2.1 mm, 1.9μm) using a 16-min linear gradient at a flow rate of 0.2 mL/min. The eluents for the positive polarity mode were eluent A (0.1% FA in Water) and eluent B (Methanol). The eluents for the negative polarity mode were eluent A (5 mM ammonium acetate, pH 9.0) and eluent B (Methanol). The solvent gradient was set as follows: 2% B, 1.5 min; 2-100% B, 12.0 min; 100% B, 14.0 min;100-2% B, 14.1 min;2% B, 16 min. Q Exactive HF-X mass spectrometer was operated in positive/negative polarity mode with spray voltage of 3.2 kV, capillary temperature of 320°C, sheath gas flow rate of 35 arb and aux gas flow rate of 10 arb.

1. **Gene expression analyses**

Total RNA from the rhizosphere roots of tobacco was isolated from 0.5g using TRIzol reagent (TaKaRa, Dalian, China) according to the manufacturer’s instructions. The ratio of absorbance at 260/280 nm was used to analyze the quality of RNA. Total RNA (2 mg) with A260/A280 ratios between 1.8 and 2.0 was used for cDNA synthesis using PrimeScript™ first strand cDNA synthesis kit (TAKARA, Bio Inc. Dalian, China). The cDNA was diluted five times with nucleasefree water (Invitrogen, Shanghai, China). The related genes were identified in this study using the National Center for Biotechnology Information (NCBI, www.ncbi.nlm.nih.gov). On the other hand, the gene primers originated from previous literature. The SYBR Green PCR Master Mix (Applied Biosystems, Singapore) was used for real-time PCR analysis. Real-time PCR analysis was performed in an optical 96-well plate using an ABI Prism 7500 (ABI company, Singapore) sequence detection system and software (PE Applied Biosystems) according to the manufacturer's instructions.

**4. Data analysis**

The raw data files generated by UHPLC-MS/MS were processed using the Compound Discoverer 3.0 (CD 3.0, Thermo Fisher) to perform peak alignment, peak picking, and quantitation for each metabolite. The main parameters were set as follows: retention time tolerance, 0.2 minutes; actual mass tolerance, 5ppm; signal intensity tolerance, 30%; signal/noise ratio, 3; and minimum intensity ,100000. After that, peak intensities were normalized to the total spectral intensity. The normalized data was used to predict the molecular formula based on additive ions, molecular ion peaks and fragment ions. And then peaks were matched with the mzCloud (https://www.mzcloud.org/) and ChemSpider (http://www.chemspider.com/) database to obtained the accurate qualitative and relative quantitative results.
